# Prenatal Whole-Genome Sequencing for Fetal Anomalies: Diagnostic Performance, Challenges, and Clinical Implications

**DOI:** 10.3390/ijms27083568

**Published:** 2026-04-16

**Authors:** Threebhorn Kamlungkuea, Kuntharee Traisrisilp, Suchaya Luewan, Jeerawan Klangjorhor, Duangrurdee Wattanasirichaigoon, Fuanglada Tongprasert

**Affiliations:** 1 Fetal Center, Faculty of Medicine, Chiang Mai University, Chiang Mai 50200, Thailand; threebhorn.k@cmu.ac.th (T.K.); kuntharee.t@cmu.ac.th (K.T.); suchaya.l@cmu.ac.th (S.L.); 2 Department of Obstetrics and Gynecology, Faculty of Medicine, Chiang Mai University, Chiang Mai 50200, Thailand; 3 Center of Multidisciplinary Technology for Advanced Medicine (CMUTEAM), Faculty of Medicine, Chiang Mai University, Chiang Mai 50200, Thailand; jeerawan.klangjorhor@cmu.ac.th; 4 Division of Medical Genetics, Department of Pediatrics, Faculty of Medicine Ramathibodi Hospital, Mahidol University, Bangkok 10400, Thailand

**Keywords:** whole-genome sequencing, fetal anomalies, congenital abnormalities, prenatal diagnosis, genetic testing, next-generation sequencing

## Abstract

Prenatal whole-genome sequencing (WGS) is a comprehensive genetic test for fetal anomalies, enabling simultaneous detection of aneuploidies, copy number variants (CNVs), single-nucleotide variants (SNVs), small insertions/deletions, structural variants, and regions of absence of heterozygosity. However, its clinical performance, optimal sequencing strategies, and implementation challenges remain incompletely defined. We conducted a narrative review of PubMed-indexed studies (1966–December 2025) evaluating prenatal WGS in fetuses with structural anomalies. Across 29 studies, diagnostic yield ranged from approximately 20% to 40%, influenced by phenotype complexity, sequencing depth, and study design. Low-coverage WGS (≤5×) reliably detected large chromosomal abnormalities with a performance comparable to chromosomal microarray analysis. Moderate-coverage WGS (20–40×) additionally enabled detection of SNVs and structural variants, providing up to 30% incremental diagnostic yield after uninformative standard testing. Turnaround times were typically 14–21 days. Higher sequencing depth increases detection of variants of uncertain significance (0.6% to 35.7%) and secondary/incidental findings (1.6–30.8%). Prenatal WGS offers meaningful diagnostic value but requires careful patient selection, multidisciplinary expertise, and structured pre- and post-test genetic counseling to ensure responsible integration into routine clinical practice, with careful consideration of clinical benefit and economic feasibility.

## 1. Introduction

Globally, congenital anomalies affect 3–6% of births annually [1,2]. EUROCAT reports a prevalence of 23.9 per 1000 births, with most cases resulting in live births, though early neonatal death and stillbirth remain notable [3]. In 2021, congenital anomalies were the third leading cause of disability-adjusted life years (DALYs) among individuals under 20 years, underscoring their substantial and sustained public health impact [1,3]. Beyond early mortality, surviving children frequently experience long-term morbidity, often requiring prolonged medical care and encountering significant social and educational challenges.

During pregnancy, prenatal ultrasonography can detect congenital anomalies and help assess their severity and likely postnatal impact. However, detection rates vary widely, from 50% in routine screening settings to 80–100% in specialized referral centers, depending on the type of anomaly, gestational age, and level of expertise [4]. Moreover, beyond structural defects, many functional organ impairments cannot be detected by prenatal ultrasonography. These functional abnormalities, which are often not apparent prenatally, may present after births as hypotonia, seizures, feeding intolerance, and neurodevelopmental delay or intellectual disability. Such postnatal phenotypes are frequently associated with genetic etiologies, which are estimated to underlie approximately 20–30% of congenital anomalies [5,6,7,8].

Genetic etiologies underlying congenital anomalies encompass a broad spectrum, including chromosomal abnormalities (aneuploidies and structural rearrangements), copy number variants (CNVs), single-gene disorders (SNVs and small indels), regions of absence of heterozygosity (AOH), non-coding regulatory variants, mosaicism, and short tandem repeats (STRs)/nucleotide repeat expansion disorders, with frequencies varying by anomaly type and population [6,9,10,11]. Establishing a genetic diagnosis can improve prognostic assessment and guide prenatal management, including reproductive decision-making, consideration of in utero therapy, delivery planning, and neonatal care, with potential to reduce neonatal morbidity and mortality. It also enables more accurate recurrence risk estimation and support genetic counseling for future reproductive options such as preimplantation genetic testing or diagnostic prenatal testing [12].

**Figure 1:**
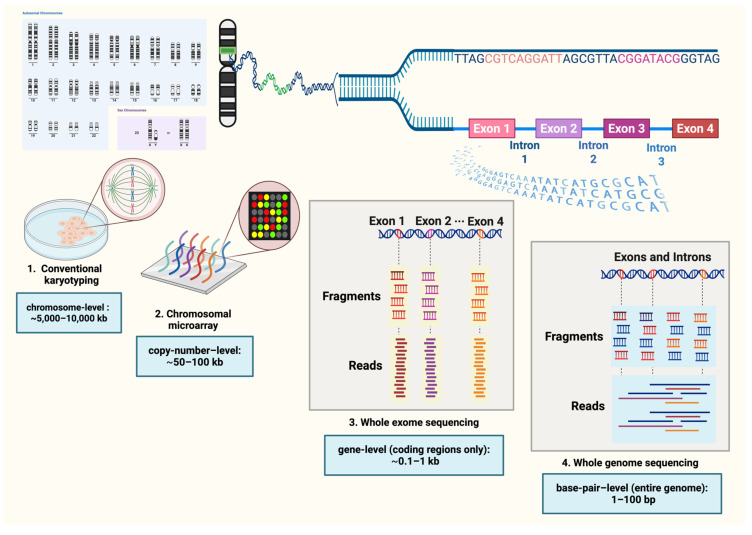
Prenatal genetic testing by techniques and resolution. Created in BioRender. ruankham, P. (2026) https://BioRender.com/jpisaec, accessed on 1 March 2026.

Prenatal genetic testing has evolved from conventional karyotyping in the late 1950s, through the introduction of chromosomal microarray analysis (CMA) in the early 2000s, to next-generation sequencing (NGS) in the following decade [13,14,15]. As technology advanced, the resolution of detectable genetic abnormalities increased, enabling identification of smaller and more complex variants (Figure 1). Conventional karyotyping typically requires 2–3 weeks of cell culture to obtain dividing cells, followed by microscopic analysis of chromosomes at the mitotic metaphase to detect aneuploidies and large structural chromosomal abnormalities, with an overall diagnostic rate of approximately 5–15% [14,16]. CMA using the oligonucleotide hybridization technique, particularly single-nucleotide polymorphism (SNP)-based arrays, enabled the detection of sub-microscopic CNVs and AOH, adding about 6–10% [14]. NGS further transformed prenatal diagnostics: targeted sequencing and whole-exome sequencing (WES), introduced in the early 2010s, increased diagnostic yield by an additional 8–15% among cases with nondiagnostic CMA results [14,17,18]. Consequently, the current standard approach is a stepwise strategy, with CMA as the first-tier test, followed by WES when CMA is uninformative [19,20].

Despite the widespread use of the stepwise approach, this two-platform workflow has limitations. While CMA captures aneuploidies/CNVs and WES detects coding SNVs (which represent approximately 80% of pathogenic variants), it has limitations in detecting other classes of genetic abnormalities including structural variants (SVs) and variants in non-coding regions [21]. It also requires more fetal DNA and may increase turnaround time and technical complexity. In contrast, WGS enables comprehensive genomic assessment on a single platform, allowing simultaneous detection of SNVs, small insertions/deletions (indels), CNVs, aneuploidies, and SVs such as inversions, translocations, and other complex rearrangements [9,22,23]. However, data on the accuracy and clinical utility of prenatal applications remain limited. A focused narrative review of its technological framework, analytical performance, and interpretive challenges is therefore essential to define its strengths, limitations, and appropriate role in guiding further comprehensive studies and its integration into routine clinical practice.

## 2. Scope and Method

To address the challenge associated with the use of prenatal WGS in fetuses with congenital anomalies, this narrative review focuses on five main areas: (1) prenatal WGS workflows, (2) prenatal WGS strategies, (3) accuracy and diagnostic yield of prenatal WGS, (4) variant detection according to sequencing coverage depth, and (5) clinical implications and challenges of prenatal WGS.

This comprehensive narrative review was conducted using the PubMed database, covering publications from 1966 through December 2025. The search strategy employed combinations of the following keywords: “prenatal”, “antenatal”, “fetal”, “fetus”, “whole-genome sequencing”, “next-generation sequencing”, “congenital”, “defect”, “disease”, “abnormalities”, “anomaly”, “malformation”, “genetic”, and “gene”. The search identified 29 original studies (comprising 7604 cases) that specifically evaluated prenatal WGS, encompassing a spectrum of sequencing resolutions and coverage depths, including low-pass, low-coverage, medium-coverage, and high-coverage WGS. Prenatal NGS-based WES was not included. For studies reporting both prenatal and postnatal sequencing, only prenatal data were extracted and analyzed. All eligible articles meeting the predefined inclusion criteria were subsequently incorporated into this review.

## 3. Results

### 3.1. Prenatal WGS: Workflows

Following prenatal diagnostic procedures including chorionic villus sampling, amniocentesis, or fetal blood sampling (cordocentesis), fetal DNA is obtained for prenatal WGS, which is performed through four main steps: (1) DNA extraction and fragmentation, (2) library preparation, (3) sequencing, and (4) bioinformatic analysis.

First, genomic DNA is extracted from prenatal specimens. The yield and integrity of fetal DNA may vary depending on specimen type, gestational age, and processing conditions. Therefore, optimized sample handling and preparation are critical to ensure adequate DNA quantity and quality for downstream sequencing analysis. The extracted DNA is subsequently fragmented into short segments, followed by assessment of maternal cell contamination to ensure fetal DNA purity and analytical reliability. Second, library preparation is performed through a series of enzymatic steps, including end repair, A-tailing, adapter ligation, and PCR amplification, during which sequencing adapters are ligated to DNA fragments to enable platform-specific sequencing [24,25]. The amplified products are then processed according to platform-specific protocols. Depending on the sequencing technology, additional steps such as denaturation, circularization, or amplification may be applied, and residual linear DNA may be removed enzymatically. Third, sequencing is performed, which may generate single-end or paired-end reads with variable read lengths depending on the technology employed [26]. The DNA fragments are fixed on a flow cell, clonally amplified, and sequenced through the fluorescently labeled nucleotides, which are detected by high-resolution imaging to determine the nucleotide sequence. Finally, bioinformatic analysis is performed. Raw sequencing data are processed through a series of steps: read alignment, variant calling, annotation, filtering, and prioritization [27]. Sequence reads are aligned to the human reference genome, followed by the identification of genetic variants, including SNVs, CNVs, SVs, and other variant classes [28]. Unlike WES, which primarily targets coding regions and is optimized for the detection of SNVs and small indels, WGS is designed to detect a boarder spectrum of genetic abnormalities simultaneously across the entire genome. The principal strength of WGS therefore lies not only in its sequencing coverage, but also in the integration of multiple bioinformatic signals within a unified analytical pipeline. From a single aligned BAM file, diverse computational signals are integrated: base-by-base comparisons for SNVs and small indels, read-depth analysis for CNVs, and paired-end mapping together with split-read information for SVs. This integrated approach enables the simultaneous detection of SNVs, CNV, and SVs within a single assay [29,30,31]. Analytical interpretation accounts for factors that may influence allele fractions, such as confined placental mosaicism, maternal contamination, and multiple gestations. Variants are filtered using population databases, inheritance models, and predicted functional impact, and subsequently prioritized based on clinical phenotype–genotype matching, integrating prenatal ultrasound findings with established databases such as OMIM and the Human Phenotype Ontology (HPO)**.** Accurate and detailed prenatal ultrasound phenotyping is critical at this stage, as incomplete or imprecise phenotypic characterization can hinder genotype–phenotype interpretation and increase the likelihood of variants of uncertain significance (VUS) or an unidentified causative variant [32]. Ultimately, identified variants are classified as pathogenic, likely pathogenic, VUS, likely benign, or benign in accordance with international ACMG/AMP guidelines [33,34,35,36].

### 3.2. Prenatal WGS Strategies: Read Length, Sequencing Depth and Variant Detection

**Figure 2:**
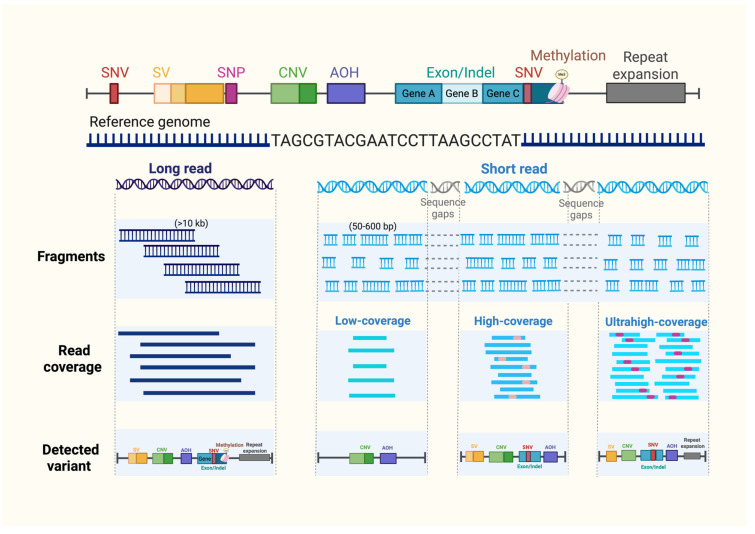
Comprehensive variant detection across sequencing platforms and coverage depths. Schematic illustration of genomic variant classes, including SNVs, SVs, SNPs, CNVs, AOH, exon-level variants, DNA methylation, and repeat expansions, mapped to the reference genome. Long-read sequencing (>10 kb) provides contiguous coverage and enhanced resolution of structural variants, repeat expansions, and methylation patterns. Short-read sequencing (50–600 bp) generates fragmented reads with potential sequence gaps but supports scalable high-depth sequencing. Increasing coverage depth (low, high, ultra-high) improves variant detection sensitivity. Abbreviations: AOH, absence of heterozygosity; bp, base pairs; CNV, copy number variant; kb, kilobase pairs; SNP, single-nucleotide polymorphism; SNV, single-nucleotide variant; SV, structural variant; VUS, variant of uncertain significance. Created in BioRender. ruankham, P. (2026) https://BioRender.com/6ji2jas, accessed on 1 March 2026.

The utility and clinical application of WGS for maximizing diagnostic benefit are determined primarily by sequencing read-length platforms and sequencing depth strategies. Sequencing read length refers to the number of base pairs generated in a single sequencing read from a DNA or RNA fragment, and current sequencing platforms are broadly classified into short-read and long-read technologies. Long-read sequencing platforms can generate reads ranging from 10,000 to over 100,000 base pairs, whereas short-read sequencing typically produces reads of up to 500–600 base pairs (Figure 2) [37,38]. Longer read lengths provide greater genomic context, enhanced improved resolution of complex genomic regions and facilitating detection of diverse classes of genetic abnormalities, including SNVs, CNVs, AOH, mosaic variants, and mitochondrial mutations. Long-read sequencing offers distinct advantages for genome assembly, as well as for the detection of SVs, STRs/nucleotide repeat expansions, and other complex genomic rearrangements. Additionally, some long-read platforms may support epigenetic analyses, such as DNA methylation [39,40].

Despite these advantages, short-read sequencing with optimized sequencing depth remains the predominant approach in current prenatal clinical practice due to its lower cost, established analytical reliability and practical implementation. This preference reflects the primary clinical objective of prenatal genetic testing: the reliable identification of causative pathogenic variants, rather than the identification of all possible genomic abnormalities. Consequently, sequencing depth (coverage) is often more critical than extended sequence context for clinical diagnostics.

Sequencing depth refers to the number of independent reads covering each base pair, and increased depth directly enhances variant detection accuracy and confidence. Large-scale genomic abnormalities, such as aneuploidies and large CNVs, can be reliably detected using low-coverage sequencing [40,41]. In contrast, accurate identification of SNVs and small insertions or deletions requires higher read depth to distinguish true variants from sequencing noises or errors, typically achieved with medium to high-coverage whole-genome sequencing (≥30×) [24,42]. In the prenatal setting, where detected variants are directly critical for clinical decisions, whether pregnancy continuation or termination, diagnostic confidence and reliability driven by the depth of sequencing are paramount. Accordingly, while long-read sequencing provides valuable genomic context, adequate sequencing depth in short-read-based prenatal WGS remains the primary determinant of diagnostic accuracy, particularly for the accurate detection of CNVs, SNVs, SVs, AOH and low-level mosaicism, which are commonly associated with congenital anomalies [37].

### 3.3. Variant Detection Performance Across Sequencing Coverage Depths in Prenatal WGS

The overall short-read prenatal WGS performance varies widely, ranging from approximately 7.5% to 100%, with an overall clinical diagnostic yield of approximately 20–40%, depending on study design, sequencing depth, patient characteristics, and the spectrum of detectable variant classes [42,43]. This variability underscores the central importance of sequencing depth in shaping the clinical utility of prenatal WGS (Table 1). However, considerable heterogeneity in study design, population characteristics, prior testing strategies, research objectives, methodologies, and specimen types contributes to this wide variation. Accordingly, reported detection rates should be interpreted with caution and within the context of individual study design. For clarity, results are categorized according to sequencing depth.

Low-coverage prenatal WGS (≤5×) can reliably detect large-scale genomic abnormalities, including CNVs, AOH, and may detect mosaic chromosomal abnormalities at moderate levels (e.g., ≥10–20% mosaic fraction) [44,45,46,47]. Low-pass or low-coverage approaches (0.25–0.5×) have reported detection rates of 7.5% to 43% [41,44,45,46,47,48]. As sequencing depth increases from 3× to >5×, detection rates increase to approximately 20–60%, reflecting improved sensitivity for sub-chromosomal abnormalities [49,50,51,52,53].

At moderate-coverage WGS (about 20–40×), the detectable variant spectrum expands to SNVs, small insertions and deletions, and SVs, in addition to CNVs and AOH. This expansion is accompanied by a clinically meaningful increase in diagnostic yield to approximately 25–40%, indicating that this depth range represents a critical threshold for comprehensive variant detection in prenatal diagnostics [24,42,43,54,55,56]. Moreover, several studies have demonstrated that prenatal WGS provides an additional diagnostic yield of up to 30% following uninformative standard karyotyping or CMA [55,57].

However, increasing sequencing depth is also associated with higher rates of variants of VUS, with reported rates reaching up to 35.7% [55]. This observation highlights an important trade-off between maximizing diagnostic yield and increasing the complexity of variant interpretation, a particularly salient issue in the prenatal setting where clinical decisions are time-sensitive and ethically complex.

Consistent with depth-dependent performance, early prenatal WGS studies largely using low-pass or low-coverage sequencing strategies demonstrate diagnostic performance comparable to standard cytogenetic approaches, including CMA and karyotyping. For example, Qi et al. (2018), Dong et al. (2016), and Walker et al. (2019) report similar detection rates between low-coverage WGS and CMA, particularly for large-scale genomic abnormalities such as aneuploidies, CNVs, and low-level mosaicism [41,44,45].

**Table 1:** Diagnostic accuracy of short-read prenatal whole-genome sequencing in fetuses with congenital anomalies.

Author	Year	Population (Fetuses)	Type of Specimen	Methodology	Singleton (S)/Trio (T)	Coverage Depth	Sample Size (Cases)	Detection Rate (%)	Detected Variant	Rate of VUS (%)	Turnaround Time (Days)	Comparative Performance vs. Standard Methods
Walker L [44]	2019	Structural anomalies	AF, CVS	WGS vs. CMA	S	Low coverage	40	7.5	CNVs, mosaicism	0	NR	Comparable
Qi H [45]	2018	Miscarriage	POC	WGS vs. CMA and karyotype	S	Low coverage	149	43.0	Aneuploidy, CNVs, mosaicism	NR	NR	Comparable
Wang H [46]	2020	PND	AF, CB, CVS	WGS vs. CMA	S	~0.25×	1023	13.5	CNVs, mosaicism	5.2	NR	Higher
Dong Z [41]	2016	Miscarriage, stillbirths	POC	WGS vs. CMA	S	~0.25×	384	41.4	CNVs, mosaicism	NR	10	Comparable
Chau MHK [47]	2020	PND, miscarriage	AF, CB, CVS, POC	WGS vs. CMA	NR	~0.25×	429	27.3	CNVs, AOH, mosaicism	0.6	3	Higher
Yang Y [48]	2022	CNS anomalies	AF, CVS	Two step WGS (0.5× → 40×)	S	~0.5×	162	38.3	SNVs, small indels, CNVs, SVs, mosaicism	NR	21	Higher
Yin Y [49]	2025	PND	AF	WGS vs. CMA	S	3×	200	21.0	CNVs, SVs, low-level mosaicism	2.0	NR	Higher
Pang J [53]	2025	Structural anomalies, PND	AF	WGS vs. CMA	S	~5×	42	61.9	CNVs, AOH, mosaicism	NR	NR	Comparable
Chang J [50]	2025	Chromosomal mosaicism detected by CMA/SNP	AF, CB, CVS	WGS vs. CMA and karyotype/FISH	NR	~5×	34	100.0 *	Aneuploidy, CNVs, AOH, low-level mosaicism	2.9	NR	Comparable
Jiang Y [52]	2025	Structural anomalies, PND	AF	WGS vs. CMA and karyotype/FISH	S	≥5×	3973	24.7	CNVs, AOH, low-level mosaicism	15.7	NR	Higher
Lü Y [51]	2023	Suspected chromosomal abnormalities, including AOH	AF, POC	WGS with AOH-specific bioinformatic algorithm	S	≥5×	24	33.3	AOH, mosaicism	NR	NR	Enable to detect prenatal AOH
Hu P [58]	2023	Structural anomalies	AF	WGS vs. CMA	Both	≥30×	185	16.8	SNVs, small indels, CNVs, STRs	NR	21–28	Higher
Wang Y [54]	2022	Structural anomalies	AF, CVS, fetal tissue	Single-step WGS vs. stepwise QF-PCR → CMA	S	>30×	37	19.0	SNVs, small indels, small CNVs	NR	NR	Higher
Westenius E [43]	2024	Structural anomalies	AF, CVS, fetal tissue	Uninformative QF-PCR and CMA → WGS	T	>30×	50	26.0	SNVs, small indels	NR	14–21	Higher
Gao Z [42]	2026	Structural anomalies	AF, CB, CVS	Single-step WGS vs. stepwise CNV-seq → WES	T	>30×	96	34.4	SNVs, small indels, CNVs, SVs, AOH, mosaicism	6.2	21	Higher
So PL [55]	2022	Structural anomalies	AF, CB, CVS, POC, fetal tissue	Uninformative QF-PCR and CMA → WGS	T	≥30×	14	35.7	SNVs, small indels, CNVs, AOH	35.7	19.5	Higher
Fu F [59]	2022	Prenatally detected BCAs	AF	WGS vs. karyotype	S	≥30×	21	81.2 **	SVs, non-coding variant	NR	NR	Enables identification of BCAs and gene disruptions
Qi Q [60]	2024	Structural anomalies	AF, CVS	Uninformative CMA and WES → WGS	T	>40×	17	11.8	SNVs, small CNVs	NR	14	Higher
Zhou J [24]	2021	Structural anomalies	AF, CB, CVS	WGS vs. CMA	T	≥40×	111	19.8	SNVs, small indels, CNVs, SVs	NR	12–24	Higher
Miceikaite I [56]	2023	Structural anomalies or NT ≥ 5 mm	AF, CVS	WGS vs. CMA	T	46.8	14	42.9	Aneuploidy, CNVs, SNVs, low-level mosaicism	NR	14	Higher
Lioa Y [61]	2022	Abnormal Sylvian fissure	AF, CB	WGS	NR	Deep WGS	28	57.1	Aneuploidy, CNVs, SNVs	NR	NR	Higher

* Concordance with CMA/SNP for mosaicism detection (validation cohort) ** Breakpoint detection in non-N-masked regions among karyotype-identified BCAs. Abbreviations: AF, amniotic fluid; AOH, absence of heterozygosity; BCAs, balanced chromosomal abnormalities; CB, umbilical cord blood; CMA, chromosomal microarray analysis; CNVs, copy number variants; CNS, central nervous system; CVS, chorionic villus sampling; FISH, fluorescence in situ hybridization; kb, kilobase pairs; NR, not reported; NT, nuchal translucency; PND, invasive prenatal diagnosis (high-risk NIPT, abnormal ultrasound, advanced maternal age, or maternal request); POC, placental tissue/product of conception; QF-PCR, quantitative fluorescent polymerase chain reaction; SNVs, single-nucleotide variants; SVs, structural variants; STRs, short tandem repeats; VUS, variants of uncertain significance; WES, whole-exome sequencing; WGS, whole-genome sequencing.

Together, these data support the feasibility of low-coverage WGS as an alternative first-tier test with comparable diagnostic yield and genome-wide scope [41,44,45].

In contrast, more recent prenatal WGS cohorts have demonstrated improved diagnostic performance relative to standard testing methods, largely driven by increased sequencing depth and more sophisticated bioinformatic pipelines. Studies by Wang et al. (2022), Yang et al. (2022), Hu et al. (2023), Jiang et al. (2025), and Gao et al. (2026) report higher diagnostic yields using moderate- to high-coverage WGS, enabling detection of a broader spectrum of variant classes, including SNVs, small insertions and deletions, SVs, AOH, and low-level mosaicism [42,48,52,54,58]. These data suggest that the diagnostic advantage of prenatal WGS becomes increasingly evident as sequencing depth and analytical capability improve as summarized in Table 2.

High-coverage sequencing (60–100×), which enables detection of SVs, STRs, and intronic or non-coding variants, has been predominantly investigated in postnatal settings, including pediatric genetic disorders and oncology, where increased sensitivity for low-frequency variants is often required [62,63]. Ultra-high sequencing depths (≥100×), which allow detection of a broader spectrum of genetic abnormalities compared with lower-coverage strategies, are generally reserved for specialized applications, such as the detection of low-fraction circulating tumor DNA, characterization of somatic mosaicism in neurodevelopmental disorders, or studies of clonal hematopoiesis and aging, rather than for routine prenatal diagnostics [64,65,66]. Consequently, despite its theoretical advantages, ultra-high-coverage WGS currently has limited clinical applicability in the prenatal setting due to higher costs, longer turnaround times, and a substantially increased interpretive burden.

Collectively, these findings indicate that the comparative diagnostic performance of prenatal WGS has evolved over time, transitioning from equivalence with conventional cytogenetic methods in early low-coverage studies to superior diagnostic utility in more recent high-coverage cohorts. Importantly, improvements in turnaround time, now typically ranging from approximately 14 to 21 days, further enhance the clinical utility of prenatal WGS by enabling timely and informed pregnancy-related decision-making [11,42,57].

### 3.4. Accuracy and Diagnostic Yield of WGS Across Anomaly Subgroups

Beyond sequencing platforms and analytical strategies, the diagnostic yield of prenatal WGS varies substantially across clinical subgroups, an important consideration for optimizing its clinical application (Table 3). Across published cohorts, prenatal WGS demonstrates an overall clinical diagnostic yield of approximately 20–40%. In a meta-analysis of 18 cohort studies, of which 8 (44%) included prenatal studies, genome sequencing provided an additional diagnostic yield of 26% over CMA following uninformative standard genetic testing [67].

To date, no large-scale meta-analysis has specifically evaluated prenatal WGS diagnostic yield stratified by phenotypic subgroups. As a result, current evidence relies largely on systematic reviews and meta-analyses of prenatal WES which provide valuable benchmarks for understanding phenotype-specific diagnostic performance in prenatal genetic testing. Across prenatal sequencing studies, diagnostic yield is consistently higher in fetuses with multiple congenital anomalies compared with those with isolated anomalies, underscoring the importance of phenotypic complexity in patient selection for prenatal WGS. In fetuses with isolated structural anomalies, prenatal genomic sequencing yields a diagnosis in approximately 10–25% of cases [43,54]. By contrast, diagnostic yield increases substantially in fetuses with multiple congenital anomalies, reaching approximately 20–40%, consistent with a higher probability of an underlying monogenic or syndromic etiology [43,68]. Although prenatal WGS is still used more often in research than in routine clinical practice, emerging studies demonstrate marked variability in diagnostic yield across different phenotypic subgroups.

In fetuses with congenital heart defects, the incremental diagnostic yield of prenatal WGS over standard testing is estimated at approximately 8–30%, with substantially higher yields observed when extracardiac anomalies are present—a pattern consistent with postnatal and infant cohorts reporting yields of up to 45% [69,70,71]. In fetuses presenting with increased nuchal translucency (≥3.5 mm) or non-immune hydrops fetalis, prenatal WGS has demonstrated diagnostic yields ranging from 32 to 52%**,** highlighting its potential value in these high-risk presentations [26,57]. For central nervous system (CNS) anomalies, reported incremental diagnostic yields range from 38 to 57%, with consistently higher yields observed in non-isolated CNS abnormalities compared with isolated findings [48,72]. Musculoskeletal anomalies represent the highest-yield subgroup, with reported diagnostic yields ranging from approximately 70–90%, reflecting the strong genetic contribution to skeletal dysplasia and related disorders [72,73].

**Table 3:** Diagnostic yield of WGS across anomaly subgroups.

System	Author	Year	GA (Weeks)	Coverage Depth	Reported Diagnostic Yield (%)	Detected Variant
Cardiovascular (8–46%)	Li J [69]	2025	20–25	5–8×	8.5	Aneuploidy, CNVs
	Cao Y [70]	2022	12–25	≥30×	30.8	SNVs, small indels, CNVs, SVs, low-level mosaicism
	Sweeney NM [71]	2021	Not reported	≥40×	45.8	SNVs, small indels, CNVs
Thick NT/hydrops fetalis (32–52%)	Choy KW [26]	2019	Not reported	≥30×	32.0	SNVs, small indels, large and small CNVs, SVs, mosaicism
	Westenius E [57]	2022	13–37	Not reported	52.2	SNVs, small indels, large and small CNVs, SVs, STRs
Central nervous system (38–57%)	Yang Y [48]	2022	Not reported	~0.5×	38.3	SNVs, small indels, CNVs, SVs, mosaicism
	Liao Y [61]	2022	21–30	Deep WGS	57.1	Aneuploidy, CNVs, SNVs
Skeletal dysplasia (70–90%)	Liu Y [72]	2019	15–30	20–30×	70	SNVs, small indels, CNVs
	Hammarsjö A [73]	2021	18–33	Not reported	89.7 *	SNVs, small indels, CNVs

* Overall diagnostic yield was calculated from 29 probands (21 WGS, 8 WES), with 26 cases showing pathogenic or likely pathogenic variants. Abbreviations: CNVs, copy number variants; NT, nuchal translucency; SNVs, single-nucleotide variants; SVs, structural variants; STRs, short tandem repeats.

Despite these encouraging yields, most prenatal WGS evidence still comes from research cohorts with stringent case selection and expert-led interpretation. Accordingly, translating prenatal WGS into routine clinical care requires standardized workflows, multidisciplinary expertise, and robust pre- and post-test genetic counseling to maximize clinical benefit while addressing interpretive complexity, uncertainty and ethical considerations.

## 4. Clinical Implications and Challenges of Prenatal WGS

Prenatal WGS demonstrates diverse performance depending on sequencing depth, resulting in variable clinical implications. Low-pass or low-coverage WGS, which has been developed and applied over a longer period, is supported by meta-analytical evidence showing that genome-wide sequencing provides incremental diagnostic yield compared with standard CMA [67]. Consequently, low-pass WGS commonly referred to as CNV sequencing has now been widely adopted as a first-tier test for the detection of chromosomal abnormalities [74,75].

For medium- to high-coverage prenatal WGS, several well-designed comparative parallel studies have evaluated its performance against standard genetic testing. Studies by Wang et al. (2022), Hu et al. (2023), and Miceikaitė et al. (2023) consistently demonstrate that prenatal WGS achieves comparable or higher diagnostic performance than CMA, particularly through its ability to detect both copy-number and sequence-level variants with a single test [54,56,58]. Although, current evidence is limited by a lack of well-designed head-to-head comparative studies between WES and WGS in prenatal populations. However, there are studies that compare short-read WGS with CMA plus WES in a prenatal setting and WGS with WES in a postnatal setting. In comparison, WGS detected all pathogenic variants identified by CMA plus WES, yielding a diagnostic rate of 19.8% (22/110) and, in addition, provided clinically significant findings, including one case of a balanced translocation that may not be detectable by CMA or WES [24]. A recent systematic review and meta-analysis demonstrates a slight increase in diagnostic yield of WGS compared with WES in postnatal cases with suspected monogenic disorders: 38.6% (95% CI, 32.6–45.0) vs. 37.8% (95% CI, 32.9–42.9) [76]. More recently, Gao et al. (2026) showed that trio-based prenatal WGS provides diagnostic performance comparable to a stepwise strategy of CNV sequencing followed by WES, while offering several practical advantages [42]. These include a more comprehensive genomic assessment, consolidation of laboratory workflows into a single test, and a shorter overall turnaround time (approximately 21 days) [42,77]. Together, these findings support the technical feasibility and diagnostic robustness of medium- to high-coverage prenatal WGS.

However, despite these advantages, increased sequencing depth generates a substantially larger volume of genomic data, thereby amplifying the complexity of bioinformatic analysis, variant interpretation, and downstream clinical decision-making. In the prenatal setting where results directly inform time-sensitive and ethically complex management decisions, the clinical utility of medium- to high-coverage prenatal whole-genome sequencing remains largely confined to research environments or highly specialized clinical centers.

### 4.1. Phenotype–Genotype Correlation and Bioinformatic Complexity

Prenatal WGS is inherently a phenotype-driven test. However, comprehensive characterization of the fetal phenotype before birth remains challenging. In most cases, the primary source of phenotypic information is routine prenatal ultrasonography. The accuracy and completeness of fetal imaging are influenced by multiple factors, including ultrasound equipment quality, fetal position, gestational age, maternal body habitus, and the expertise of the sonographer, all of which contribute to diagnostic uncertainty. Beyond ultrasound, additional clinical information such as fetal magnetic resonance imaging, results from prior prenatal genetic testing, detailed family history of congenital anomalies or genetic disorders, pedigree analysis, parental medical history, ethnicity, reproductive history, and consanguinity may support variant interpretation [12].

Importantly, fetal phenotypes may evolve as pregnancy progresses or become more clearly defined after birth, which can substantially affect genotype–phenotype correlation. Previous studies by Chandler et al. (2022) and Mone et al. (2022) demonstrate that among fetuses with prenatally reported VUS, 21–27% were reclassified as likely pathogenic postnatally following the emergence of additional phenotypic features [78,79]. These findings underscore the limitations of static prenatal phenotyping and the dynamic nature of variant interpretation. Furthermore, current genotype–phenotype reference resources, including widely used databases such as OMIM and the Human Phenotype Ontology, are predominantly derived from postnatal disease presentations and remain relatively under-representative of prenatal phenotypes. This gap further complicates variant prioritization and interpretation in the prenatal setting [80].

### 4.2. Unintentional Findings in Prenatal Whole-Genome Sequencing and Counseling Implications

Owing to the comprehensive genomic coverage and increasing analytical depth, WGS leads to the detection of a higher number and a wide variety of genomic abnormalities. While this enhances diagnostic potential, it also increases the likelihood of identifying unintentional findings that contribute to interpretive, ethical, and counseling challenges. Three major categories of unintentional findings require careful consideration and management: VUS, secondary findings, and incidental findings. All three issues necessitate explicit discussion during pre-test genetic counseling to support informed parental decision-making [81].

1. **Variants of uncertain significance (VUS):** Prenatal WGS is associated with a high rate of VUS, reflecting current limitations in genomic databases, incomplete genotype–phenotype correlations, and the evolving nature of variant interpretation. This challenge is particularly pronounced in the prenatal setting, where phenotyping information is often incomplete or dynamic, further complicating variant interpretation. In addition, higher sequencing depth and broader genomic interrogation may increase the likelihood of identifying variants with uncertain clinical significance. Previous studies have reported VUS rates ranging from approximately 5.2% at low coverage (~0.25×), increasing to around 15.7% at 5× coverage, and up to 35.7% at high coverage (≥30) [46,52,55]. Pre-test counseling should explicitly prepare parents for the possibility that WGS may identify variants that cannot be confidently classified as benign or pathogenic at the time of reporting and that such findings are not diagnostic. Trio-based sequencing, in which fetal and both biological parental samples are sequenced and analyzed concurrently, is strongly preferred for prenatal diagnostic sequencing, reduces the rate of uninterpretable variants, facilitates identification of de novo variants, enables determination of biallelic pathogenic variants (homozygous or compound heterozygous), and clarifies mode of inheritance, thereby improving diagnostic confidence. When proband-only (singleton) sequencing is performed, any potentially diagnostic fetal variant generally requires subsequent targeted testing of parental samples to confirm inheritance and clinical relevance [12,21].
  Several strategies may mitigate the burden of VUS in prenatal WGS, including phenotype-driven variant prioritization, restriction of analysis to clinically relevant gene sets, and the application of stringent filtering criteria. Incorporation of detailed prenatal phenotyping, together with the expansion of population-specific genomic databases, may further enhance variant interpretation. In addition, periodic reanalysis and multidisciplinary review are essential, as variant classification may evolve with accumulating genomic knowledge and the emergence of additional fetal or postnatal phenotypic data.
  Nevertheless, VUC cannot be entirely avoided, and parents should be counseled regarding the potential for future reclassification. This consideration is particularly important in the prenatal setting, where phenotypic features may evolve during gestation or become more apparent after birth, thereby refining genotype–phenotype correlations. Notably, approximately 21–27% of prenatally identified VUS are reclassified as likely pathogenic following the emergence of postnatal phenotypic features [78,79]. The identification may introduce uncertainty in clinical decision-making, especially when results inform pregnancy management. Therefore, a structured approach to VUS management, including multidisciplinary review, periodic reanalysis, and postnatal phenotypic follow-up, is essential to improve interpretation and clinical utility over time.
2. **Secondary findings (SFs)** refer to pathogenic or likely pathogenic variants identified in genes unrelated to the fetal phenotype, but which are deliberately analyzed and reported according to recommendations from the American College of Medical Genetics and Genomics (ACMG) [35,82]. These genes are selected because they are associated with medically actionable conditions, for which early detection and clinical intervention may improve outcomes in otherwise asymptomatic individuals such as cancer predisposition syndromes (e.g., hereditary breast and ovarian cancer, Lynch syndrome) or cardiovascular disorders (e.g., cardiomyopathies, inherited arrhythmia syndromes). In the prenatal setting, secondary findings should not be used to guide reproductive decision-making, such as pregnancy continuation or termination. However, ACMG guidance emphasizes that the possibility of identifying secondary findings must be discussed during pre-test counseling, and parents must be offered the option to opt in or opt out of receiving such results as part of the informed consent process [12,36,81]. Reported incidence of SFs in prenatal WGS ranges from approximately 7.7–14.3% [55,70].
3. **Incidental findings** include pathogenic or likely pathogenic variants that are unrelated to the primary testing indication, not included in the ACMG secondary findings gene list and identified unintentionally during genome-wide analysis. In the fetal context, incidental findings may involve genes associated with neurodevelopmental disorders, intellectual disability, or metabolic conditions that do not present with detectable prenatal ultrasound abnormalities. Importantly, highly penetrant pathogenic variants causing moderate-to-severe childhood onset disorders are generally recommended for reporting, as early diagnosis may have implications for postnatal management. Many of these conditions are not detectable by prenatal imaging. Incidental findings are considered ethically sensitive, particularly in pregnancy, due to their potential psychological impact and uncertain relevance to immediate prenatal care [12,81]. Reported rates of incidental findings in prenatal WGS range from approximately 1.6% to 30.8% [42,58].

Accordingly, prenatal counseling and informed consent for WGS must place particular emphasis on discussing the scope, limitations, and ethical implications of VUS, secondary findings, and incidental findings, as well as the likelihood of their detection and the potential implications for postnatal management and follow-up.

### 4.3. Economic Feasibility

Economic feasibility remains a major barrier to the clinical implementation of prenatal WGS at present, particularly when compared with the current diagnostic standard (CMA combined with WES), and this challenge is further amplified in resource-limited settings. In addition, WGS is associated with longer turnaround times, requires complex bioinformatic pipelines, and yields a substantial proportion of VUS, which may lead to inconclusive results, which is an important consideration given the time-sensitive nature of prenatal decision-making.

Recent postnatal studies in pediatric patients with neurodevelopmental disorders and in critically ill populations suggest that first-tier WGS may be cost-effective compared with stepwise approaches (e.g., CMA followed by WES), owing to improved diagnostic yield and reduced downstream healthcare utilization [83,84,85]. However, these findings cannot be directly extrapolated to the prenatal setting. Neurodevelopmental and critical illness cohorts are more likely to involve functional abnormalities, which differ fundamentally from the structural anomalies typically detected prenatally. Furthermore, postnatal evaluation allows for the identification of evolving phenotypes, including multi-organ dysfunction and dysmorphic features, which may not be apparent in utero. Therefore, dedicated studies in prenatal populations are required to determine the true cost effectiveness and clinical utility of WGS in this context.

## 5. Future Perspectives

WGS is expected to enhance diagnostic accuracy and clinical applicability through continued refinement of bioinformatic pipelines, improved detection of complex variant classes, and better integration of prenatal-specific genotype–phenotype data. Advances in sequencing technology and analytical strategies may further reduce turnaround time and costs, while enabling the identification of an optimal WGS coverage that maximizes detection of clinically relevant prenatal genetic abnormalities while maintaining a practical balance between diagnostic yield and interpretive burden in the prenatal setting.

From a clinical standpoint, broader implementation of prenatal WGS will depend on the establishment of standardized guidelines for patient selection, variant interpretation, and the management of secondary and incidental findings. Multidisciplinary collaboration and comprehensive genetic counseling will remain essential to address ethical, psychosocial, and decision-making challenges. As evidence regarding clinical utility and cost-effectiveness continues to accumulate, prenatal WGS may evolve from a predominantly research-based tool into a selectively applied clinical diagnostic option within specialized prenatal care settings.

In addition, emerging technologies, including long-read sequencing, methylation profiling, and transcriptomic analysis, hold promise for enhancing genomic interpretation in the future. However, their current application in prenatal diagnostics remains limited due to technical, cost, and feasibility considerations.

## 6. Conclusions

Prenatal WGS offers a comprehensive genomic diagnostic approach with clinically meaningful diagnostic yield in fetuses with congenital anomalies, particularly when applied at a moderate sequencing depth. Although prenatal WGS demonstrates strong theoretical performance in terms of diagnostic accuracy, incremental yield, and breadth of detectable variant classes, its clinical implementation remains challenged by interpretive complexity, unintentional findings, and ethical considerations. Careful case selection, multidisciplinary expertise, and structured pre- and post-test genetic counseling frameworks are therefore essential for the responsible integration of prenatal WGS into clinical practice, with careful consideration of clinical benefit and economic feasibility.

## Figures and Tables

**Table 2:** Variant detection according to sequencing coverage depth.

Variant Class/Category	Low Coverage (≤5×)	Moderate Coverage (20–40×)	High Coverage (60–100×)	Ultra-High Coverage (≥100×)	Long Read (30–50×)
Aneuploidy	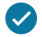	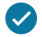	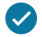	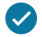	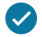
CNVs	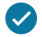	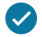	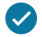	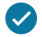	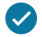
AOH	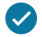	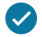	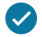	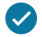	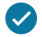
Exon deletion/duplication	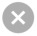	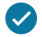	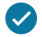	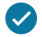	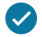
Small indels (<50 bp)	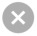	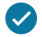	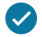	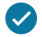	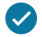
Low-level mosaicism	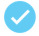	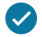	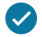	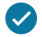	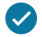
SVs	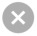	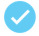	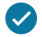	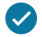	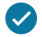
SNVs	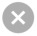	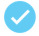	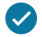	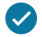	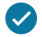
STRs/repeat expansions	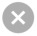	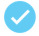	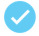	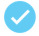	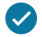
None-coding/deep intron	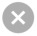	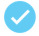	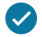	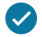	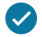
Mitochondrial variants	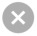	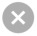	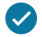	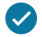	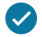
Methylation	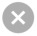	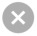	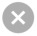	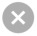	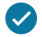
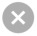 Not detectable		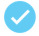 Limited detectable		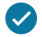 Detectable	

Abbreviations: AOH, absence of heterozygosity; CNVs, copy number variants; SNVs, single-nucleotide variants; SVs, structural variants; STRs, short tandem repeats.

## Data Availability

No new data were created or analyzed in this study. Data sharing is not applicable to this article.

## References

[1] Li Y., He C., Yu H., Wu D., Liu L., Zhang X. (2025). Global, regional, and national epidemiology of congenital birth defects in children from 1990 to 2021: A cross-sectional study. BMC Pregnancy Childbirth.

[2] Duan J., Ding R., Yu Y., Li M., Ruan Y., Hu Y., He Y., Sun Z. (2025). Global and regional burden of congenital birth defects, 1990-2021: Persistent healthcare disparities and emerging challenges from non-fatal health burden. BMJ Public Health.

[3] Loane M., Dolk H., Garne E., Greenlees R. (2011). Paper 3: EUROCAT data quality indicators for population-based registries of congenital anomalies. Birth Defects Res. A Clin. Mol. Teratol..

[4] Santoro M., Coi A., Masini G., Pasquini L. (2025). Prenatal detection rate of congenital anomalies over a period of 30 years: A population-based registry study. Eur. J. Obstet. Gynecol. Reprod. Biol..

[5] Cleper R., Kapra O., Goldental N., Gross R. (2025). The association between autism spectrum disorder and congenital malformations: A population-based nested case-control study. Mol. Psychiatry.

[6] Bale J.R., Stoll B.J., Lucas A.O., Institute of Medicine Committee on Improving Birth Outcomes (2003). Reducing Birth Defects: Meeting the Challenge in the Developing World.

[7] Rodan L.H., Stoler J., Chen E., Geleske T. (2025). Genetic Evaluation of the Child with Intellectual Disability or Global Developmental Delay: Clinical Report. Pediatrics.

[8] Wang H., Lin X., Lyu G., He S., Dong B., Yang Y. (2023). Chromosomal abnormalities in fetuses with congenital heart disease: A meta-analysis. Arch. Gynecol. Obstet..

[9] Best S., Wou K., Vora N., Van der Veyver I.B., Wapner R., Chitty L.S. (2018). Promises, pitfalls and practicalities of prenatal whole exome sequencing. Prenat. Diagn..

[10] Wojcik M.H., Agrawal P.B. (2020). Deciphering congenital anomalies for the next generation. Cold Spring Harb. Mol. Case Stud..

[11] Dong Z., Wang H., Chen H., Jiang H., Yuan J., Yang Z., Wang W.J., Xu F., Guo X., Cao Y. (2018). Identification of balanced chromosomal rearrangements previously unknown among participants in the 1000 Genomes Project: Implications for interpretation of structural variation in genomes and the future of clinical cytogenetics. Genet. Med..

[12] Monaghan K.G., Leach N.T., Pekarek D., Prasad P., Rose N.C. (2020). The use of fetal exome sequencing in prenatal diagnosis: A points to consider document of the American College of Medical Genetics and Genomics (ACMG). Genet. Med..

[13] Steele M.W., Breg W.R. (1966). Chromosome analysis of human amniotic-fluid cells. Lancet.

[14] Wapner R.J., Martin C.L., Levy B., Ballif B.C., Eng C.M., Zachary J.M., Savage M., Platt L.D., Saltzman D., Grobman W.A. (2012). Chromosomal microarray versus karyotyping for prenatal diagnosis. N. Engl. J. Med..

[15] Carss K.J., Hillman S.C., Parthiban V., McMullan D.J., Maher E.R., Kilby M.D., Hurles M.E. (2014). Exome sequencing improves genetic diagnosis of structural fetal abnormalities revealed by ultrasound. Hum. Mol. Genet..

[16] Wei S., Yuan Y., Tu S., Pang C., Chen M., Ren M. (2025). Karyotyping with amniotic fluid in 6,572 pregnant women and pregnancy outcomes—A single-center retrospective study. PLoS ONE.

[17] Lord J., McMullan D.J., Eberhardt R.Y., Rinck G., Hamilton S.J., Quinlan-Jones E., Prigmore E., Keelagher R., Best S.K., Carey G.K. (2019). Prenatal exome sequencing analysis in fetal structural anomalies detected by ultrasonography (PAGE): A cohort study. Lancet.

[18] Petrovski S., Aggarwal V., Giordano J.L., Stosic M., Wou K., Bier L., Spiegel E., Brennan K., Stong N., Jobanputra V. (2019). Whole-exome sequencing in the evaluation of fetal structural anomalies: A prospective cohort study. Lancet.

[19] American College of Obstetricians and Gynecologists (2016). Committee Opinion No. 682: Microarrays and Next-Generation Sequencing Technology: The Use of Advanced Genetic Diagnostic Tools in Obstetrics and Gynecology. Obstet. Gynecol..

[20] American College of Obstetricians and Gynecologists (2018). ACOG Technology Assessment in Obstetrics and Gynecology No. 14: Modern Genetics in Obstetrics and Gynecology. Obstet. Gynecol..

[21] Jeanne M., Chung W.K. (2025). Prenatal genomic sequencing: Navigating uncertainty. Semin. Perinatol..

[22] Stavropoulos D.J., Merico D., Jobling R., Bowdin S., Monfared N., Thiruvahindrapuram B., Nalpathamkalam T., Pellecchia G., Yuen R.K.C., Szego M.J. (2016). Whole Genome Sequencing Expands Diagnostic Utility and Improves Clinical Management in Pediatric Medicine. NPJ Genom. Med..

[23] Raca G., Astbury C., Behlmann A., De Castro M.J., Hickey S.E., Karaca E., Lowther C., Riggs E.R., Seifert B.A., Thorland E.C. (2023). Points to consider in the detection of germline structural variants using next-generation sequencing: A statement of the American College of Medical Genetics and Genomics (ACMG). Genet. Med..

[24] Zhou J., Yang Z., Sun J., Liu L., Zhou X., Liu F., Xing Y., Cui S., Xiong S., Liu X. (2021). Whole Genome Sequencing in the Evaluation of Fetal Structural Anomalies: A Parallel Test with Chromosomal Microarray Plus Whole Exome Sequencing. Genes.

[25] Talkowski M.E., Ordulu Z., Pillalamarri V., Benson C.B., Blumenthal I., Connolly S., Hanscom C., Hussain N., Pereira S., Picker J. (2012). Clinical diagnosis by whole-genome sequencing of a prenatal sample. N. Engl. J. Med..

[26] Choy K.W., Wang H., Shi M., Chen J., Yang Z., Zhang R., Yan H., Wang Y., Chen S., Chau M.H.K. (2019). Prenatal Diagnosis of Fetuses with Increased Nuchal Translucency by Genome Sequencing Analysis. Front. Genet..

[27] Huang J., Liang X., Xuan Y., Geng C., Li Y., Lu H., Qu S., Mei X., Chen H., Yu T. (2017). A reference human genome dataset of the BGISEQ-500 sequencer. Gigascience.

[28] Mao Q., Chin R., Xie W., Deng Y., Zhang W., Xu H., Zhang R.Y., Shi Q., Peters E.E., Gulbahce N. (2018). Advanced Whole-Genome Sequencing and Analysis of Fetal Genomes from Amniotic Fluid. Clin. Chem..

[29] Layer R.M., Chiang C., Quinlan A.R., Hall I.M. (2014). LUMPY: A probabilistic framework for structural variant discovery. Genome Biol..

[30] Pirooznia M., Goes F.S., Zandi P.P. (2015). Whole-genome CNV analysis: Advances in computational approaches. Front. Genet..

[31] Gabrielaite M., Torp M.H., Rasmussen M.S., Andreu-Sánchez S., Vieira F.G., Pedersen C.B., Kinalis S., Madsen M.B., Kodama M., Demircan G.S. (2021). A Comparison of Tools for Copy-Number Variation Detection in Germline Whole Exome and Whole Genome Sequencing Data. Cancers.

[32] Abou Tayoun A.N., Spinner N.B., Rehm H.L., Green R.C., Bianchi D.W. (2018). Prenatal DNA Sequencing: Clinical, Counseling, and Diagnostic Laboratory Considerations. Prenat. Diagn..

[33] Riggs E.R., Andersen E.F., Cherry A.M., Kantarci S., Kearney H., Patel A., Raca G., Ritter D.I., South S.T., Thorland E.C. (2020). Technical standards for the interpretation and reporting of constitutional copy-number variants: A joint consensus recommendation of the American College of Medical Genetics and Genomics (ACMG) and the Clinical Genome Resource (ClinGen). Genet. Med..

[34] Richards S., Aziz N., Bale S., Bick D., Das S., Gastier-Foster J., Grody W.W., Hegde M., Lyon E., Spector E. (2015). Standards and guidelines for the interpretation of sequence variants: A joint consensus recommendation of the American College of Medical Genetics and Genomics and the Association for Molecular Pathology. Genet. Med..

[35] Green R.C., Berg J.S., Grody W.W., Kalia S.S., Korf B.R., Martin C.L., McGuire A.L., Nussbaum R.L., O’Daniel J.M., Ormond K.E. (2013). ACMG recommendations for reporting of incidental findings in clinical exome and genome sequencing. Genet. Med..

[36] Miller D.T., Lee K., Gordon A.S., Amendola L.M., Adelman K., Bale S.J., Chung W.K., Gollob M.H., Harrison S.M., Herman G.E. (2021). Recommendations for reporting of secondary findings in clinical exome and genome sequencing, 2021 update: A policy statement of the American College of Medical Genetics and Genomics (ACMG). Genet. Med..

[37] Chau M.H.K., Choolani M., Dong Z., Cao Y., Choy K.W. (2024). Genome sequencing in the prenatal diagnosis of structural malformations in the fetus. Best. Pract. Res. Clin. Obstet. Gynaecol..

[38] Karst S.M., Ziels R.M., Kirkegaard R.H., Sørensen E.A., McDonald D., Zhu Q., Knight R., Albertsen M. (2021). High-accuracy long-read amplicon sequences using unique molecular identifiers with Nanopore or PacBio sequencing. Nat. Methods.

[39] Olivucci G., Iovino E., Innella G., Turchetti D., Pippucci T., Magini P. (2024). Long read sequencing on its way to the routine diagnostics of genetic diseases. Front. Genet..

[40] Basel-Salmon L., Brabbing-Goldstein D. (2024). Fetal whole genome sequencing as a clinical diagnostic tool: Advantages, limitations and pitfalls. Best Pract. Res. Clin. Obstet. Gynaecol..

[41] Dong Z., Zhang J., Hu P., Chen H., Xu J., Tian Q., Meng L., Ye Y., Wang J., Zhang M. (2016). Low-pass whole-genome sequencing in clinical cytogenetics: A validated approach. Genet. Med..

[42] Gao Z., Liu M., Jiang J., Yang X., Li Y., Zhang Y., Wang Y., Hua C., Liu N., Zhu X. (2026). Whole-genome sequencing for the prenatal evaluation of fetal structural anomalies: A prospective multicenter study. Am. J. Obstet. Gynecol..

[43] Westenius E., Conner P., Pettersson M., Sahlin E., Papadogiannakis N., Lindstrand A., Iwarsson E. (2024). Whole-genome sequencing in prenatally detected congenital malformations: Prospective cohort study in clinical setting. Ultrasound Obstet. Gynecol..

[44] Walker L., Watson C.M., Hewitt S., Crinnion L.A., Bonthron D.T., Cohen K.E. (2019). An alternative to array-based diagnostics: A prospectively recruited cohort, comparing arrayCGH to next-generation sequencing to evaluate foetal structural abnormalities. J. Obstet. Gynaecol..

[45] Qi H., Xuan Z.L., Du Y., Cai L.R., Zhang H., Wen X.H., Kong X.D., Yang K., Mi Y., Fu X.X. (2018). High resolution global chromosomal aberrations from spontaneous miscarriages revealed by low coverage whole genome sequencing. Eur. J. Obstet. Gynecol. Reprod. Biol..

[46] Wang H., Dong Z., Zhang R., Chau M.H.K., Yang Z., Tsang K.Y.C., Wong H.K., Gui B., Meng Z., Xiao K. (2020). Low-pass genome sequencing versus chromosomal microarray analysis: Implementation in prenatal diagnosis. Genet. Med..

[47] Chau M.H.K., Wang H., Lai Y., Zhang Y., Xu F., Tang Y., Wang Y., Chen Z., Leung T.Y., Chung J.P.W. (2020). Low-pass genome sequencing: A validated method in clinical cytogenetics. Hum. Genet..

[48] Yang Y., Zhao S., Sun G., Chen F., Zhang T., Song J., Yang W., Wang L., Zhan N., Yang X. (2022). Genomic architecture of fetal central nervous system anomalies using whole-genome sequencing. NPJ Genom. Med..

[49] Yin Y., He Y., Chen C., He Y., Wang J., Qin S., Wang H., Ma K., Hu D., Xiao R. (2025). A Prospective Evaluation of the Diagnostic Utility for Low-Coverage Genome Sequencing in Prenatal Samples: A Comparison With Chromosomal Microarray Analysis. Prenat. Diagn..

[50] Chang J., Li M., Jiang Y., Zhou X., Hao N., Yu Y., Lü Y., Qi Q. (2025). Prenatal diagnosis and pregnancy outcomes of mosaicism detected by CMA-seq. BMC Pregnancy Childbirth.

[51] Lü Y., Jiang Y., Zhou X., Hao N., Xu C., Guo R., Chang J., Li M., Zhang H., Zhou J. (2023). Detection of Mosaic Absence of Heterozygosity (AOH) Using Low-Pass Whole Genome Sequencing in Prenatal Diagnosis: A Preliminary Report. Diagnostics.

[52] Jiang Y., Liu F., Zhong L., Zhong R., Liang M., Ma L., Zhang V.W., Chen B., Zhang Q., Xu L. (2025). Prenatal Diagnosis of Foetal Structural Anomalies Using Medium-Coverage Whole Genome Sequencing (CMA-Seq): A Large-Scale Comparative Study With CMA in 3973 Pregnancies. BJOG.

[53] Pang J., Zhou L., Hu J., Kuang H., Xi H., Ma N., Yang S., Yu W., Zhang Y., Zhang Q. (2025). A Comparative Study of Medium-Coverage Genome Sequencing and SNP Array Technology in Identifying Chromosomal Abnormalities to Advance Prenatal and Postnatal Diagnosis. J. Mol. Diagn..

[54] Wang Y., Greenfeld E., Watkins N., Belesiotis P., Zaidi S.H., Marshall C., Thiruvahindrapuram B., Shannon P., Roifman M., Chong K. (2022). Diagnostic yield of genome sequencing for prenatal diagnosis of fetal structural anomalies. Prenat. Diagn..

[55] So P.L., Hui A.S.Y., Ma T.W.L., Shu W., Hui A.P.W., Kong C.W., Lo T.K., Kan A.N.C., Kan E.Y.L., Chong S.C. (2022). Implementation of Public Funded Genome Sequencing in Evaluation of Fetal Structural Anomalies. Genes.

[56] Miceikaite I., Fagerberg C., Brasch-Andersen C., Torring P.M., Kristiansen B.S., Hao Q., Sperling L., Ibsen M.H., Löser K., Bendsen E.A. (2023). Comprehensive prenatal diagnostics: Exome versus genome sequencing. Prenat. Diagn..

[57] Westenius E., Sahlin E., Conner P., Lindstrand A., Iwarsson E. (2022). Diagnostic yield using whole-genome sequencing and in-silico panel of 281 genes associated with non-immune hydrops fetalis in clinical setting. Ultrasound Obstet. Gynecol..

[58] Hu P., Zhang Q., Cheng Q., Luo C., Zhang C., Zhou R., Meng L., Huang M., Wang Y., Wang Y. (2023). Whole genome sequencing vs chromosomal microarray analysis in prenatal diagnosis. Am. J. Obstet. Gynecol..

[59] Fu F., Li R., Dang X., Yu Q., Xu K., Gu W., Wang D., Yang X., Pan M., Zhen L. (2022). Prenatal diagnosis of 21 fetuses with balanced chromosomal abnormalities (BCAs) using whole-genome sequencing. Front. Genet..

[60] Qi Q., Jiang Y., Zhou X., Lü Y., Xiao R., Bai J., Lou H., Sun W., Lian Y., Hao N. (2024). Whole-genome sequencing analysis in fetal structural anomalies: Novel phenotype-genotype discoveries. Ultrasound Obstet. Gynecol..

[61] Liao Y., Yang Y., Wen H., Wang B., Zhang T., Li S. (2022). Abnormal Sylvian fissure at 20–30 weeks as indicator of malformations of cortical development: Role of prenatal whole-genome sequencing. Ultrasound Obstet. Gynecol..

[62] Vavoulis D.V., Cutts A., Thota N., Brown J., Sugar R., Rueda A., Ardalan A., Howard K., Matos Santo F., Sannasiddappa T. (2025). Multimodal cell-free DNA whole-genome TAPS is sensitive and reveals specific cancer signals. Nat. Commun..

[63] Albuquerque A.L.B., Dos Santos G.G., Sadok S.H., Antonello B.B., de Jesus L.M., de Carvalho M.E.A., Mutarelli A., Ribeiro P.V.Z. (2025). Diagnostic Yield of Genome Sequencing Versus Exome Sequencing in Pediatric Patients with Rare Phenotypes: A Systematic Review and Meta-Analysis. Am. J. Med. Genet. A.

[64] Ganesamoorthy D., Robertson A.J., Chen W., Hall M.B., Cao M.D., Ferguson K., Lakhani S.R., Nones K., Simpson P.T., Coin L.J.M. (2022). Whole genome deep sequencing analysis of cell-free DNA in samples with low tumour content. BMC Cancer.

[65] Tursky M.L., Artuz C.M., Rapadas M., Wittert G.A., Molloy T.J., Ma D.D. (2025). Error-corrected ultradeep next-generation sequencing for detection of clonal haematopoiesis and haematological neoplasms—Sensitivity, specificity and accuracy. PLoS ONE.

[66] Rodin R.E., Dou Y., Kwon M., Sherman M.A., D’Gama A.M., Doan R.N., Rento L.M., Girskis K.M., Bohrson C.L., Kim S.N. (2021). The landscape of somatic mutation in cerebral cortex of autistic and neurotypical individuals revealed by ultra-deep whole-genome sequencing. Nat. Neurosci..

[67] Shreeve N., Sproule C., Choy K.W., Dong Z., Gajewska-Knapik K., Kilby M.D., Mone F. (2024). Incremental yield of whole-genome sequencing over chromosomal microarray analysis and exome sequencing for congenital anomalies in prenatal period and infancy: Systematic review and meta-analysis. Ultrasound Obstet. Gynecol..

[68] Lim K.M.X., Gibbs A., Scotchman E., Smith G., Chitty L.S., Chandler N.J. (2026). Diagnostic Yield of Sequencing for Prenatal Diagnosis of Fetal Structural Anomalies: An Updated Systematic Review. Prenat. Diagn..

[69] Li J., Zhu Y., Tian Y., Li Y., Zhi Y., Ouyang X., Yang X., Xu L., Zhang Q., Zhang W.V. (2025). Prenatal diagnostic value of medium-coverage WGS and clinical Exome sequencing in fetal congenital heart disease. Future Sci. OA.

[70] Cao Y., Chau M.H.K., Zheng Y., Zhao Y., Kwan A.H.W., Hui S.Y.A., Lam Y.H., Tan T.Y.T., Tse W.T., Wong L. (2022). Exploring the diagnostic utility of genome sequencing for fetal congenital heart defects. Prenat. Diagn..

[71] Sweeney N.M., Nahas S.A., Chowdhury S., Batalov S., Clark M., Caylor S., Cakici J., Nigro J.J., Ding Y., Veeraraghavan N. (2021). Rapid whole genome sequencing impacts care and resource utilization in infants with congenital heart disease. NPJ Genom. Med..

[72] Liu Y., Wang L., Yang Y.K., Liang Y., Zhang T.J., Liang N., Yang L.M., Li S.J., Shan D., Wu Q.Q. (2019). Prenatal diagnosis of fetal skeletal dysplasia using targeted next-generation sequencing: An analysis of 30 cases. Diagn. Pathol..

[73] Hammarsjö A., Pettersson M., Chitayat D., Handa A., Anderlid B.M., Bartocci M., Basel D., Batkovskyte D., Beleza-Meireles A., Conner P. (2021). High diagnostic yield in skeletal ciliopathies using massively parallel genome sequencing, structural variant screening and RNA analyses. J. Hum. Genet..

[74] Zheng Y., Zhu B., Tan J., Guan Y., Morton C.C., Lu G., The Chinese Genomic Structural Variants (2022). Experience of Low-Pass Whole-Genome Sequencing-Based Copy Number Variant Analysis: A Survey of Chinese Tertiary Hospitals. Diagnostics.

[75] Mazzonetto P.C., Villela D., Krepischi A.C.V., Pierry P.M., Bonaldi A., Almeida L.G.D., Paula M.G., Bürger M.C., de Oliveira A.G., Fonseca G.G.G. (2024). Low-pass whole genome sequencing as a cost-effective alternative to chromosomal microarray analysis for low- and middle-income countries. Am. J. Med. Genet. A.

[76] Nurchis M.C., Altamura G., Riccardi M.T., Radio F.C., Chillemi G., Bertini E.S., Garlasco J., Tartaglia M., Dallapiccola B., Damiani G. (2023). Whole genome sequencing diagnostic yield for paediatric patients with suspected genetic disorders: Systematic review, meta-analysis, and GRADE assessment. Arch. Public Health.

[77] Kilby M.D., Morgan S., Mone F., Williams D. (2023). Prenatal next-generation sequencing in the fetus with congenital malformations: How can we improve clinical utility?. Am. J. Obstet. Gynecol. MFM.

[78] Chandler N.J., Scotchman E., Mellis R., Ramachandran V., Roberts R., Chitty L.S. (2022). Lessons learnt from prenatal exome sequencing. Prenat. Diagn..

[79] Mone F., Abu Subieh H., Doyle S., Hamilton S., McMullan D.J., Allen S., Marton T., Williams D., Kilby M.D. (2022). Evolving fetal phenotypes and clinical impact of progressive prenatal exome sequencing pathways: Cohort study. Ultrasound Obstet. Gynecol..

[80] Liu P., Vossaert L. (2022). Emerging technologies for prenatal diagnosis: The application of whole genome and RNA sequencing. Prenat. Diagn..

[81] Van den Veyver I.B., Chandler N., Wilkins-Haug L.E., Wapner R.J., Chitty L.S. (2022). International Society for Prenatal Diagnosis Updated Position Statement on the use of genome-wide sequencing for prenatal diagnosis. Prenat. Diagn..

[82] Lee K., Abul-Husn N.S., Amendola L.M., Brothers K.B., Chung W.K., Gollob M.H., Gordon A.S., Harrison S.M., Hershberger R.E., Li M. (2025). ACMG SF v3.3 list for reporting of secondary findings in clinical exome and genome sequencing: A policy statement of the American College of Medical Genetics and Genomics (ACMG). Genet. Med..

[83] Nurchis M.C., Radio F.C., Salmasi L., Heidar Alizadeh A., Raspolini G.M., Altamura G., Tartaglia M., Dallapiccola B., Pizzo E., Gianino M.M. (2024). Cost-Effectiveness of Whole-Genome vs Whole-Exome Sequencing Among Children With Suspected Genetic Disorders. JAMA Netw. Open.

[84] Runheim H., Pettersson M., Hammarsjö A., Nordgren A., Henriksson M., Lindstrand A., Levin L., Soller M.J. (2023). The cost-effectiveness of whole genome sequencing in neurodevelopmental disorders. Sci. Rep..

[85] Sanford Kobayashi E., Waldman B., Engorn B.M., Perofsky K., Allred E., Briggs B., Gatcliffe C., Ramchandar N., Gold J.J., Doshi A. (2021). Cost Efficacy of Rapid Whole Genome Sequencing in the Pediatric Intensive Care Unit. Front. Pediatr..

